# Genetic Variants at the 9p21.3 Locus Are Associated with Risk for Non-Compressible Artery Disease: Results from the ARTPER Study

**DOI:** 10.3390/genes15010002

**Published:** 2023-12-19

**Authors:** Marc Via, Guillem Pera, Rosa Forés, Anna Costa-Garrido, Antonio Heras, José Miguel Baena-Díez, Edurne Pedrosa, Inmaculada C. Clemente, Noemí Lamonja-Vicente, Maria Mataró, Pere Torán-Montserrat, M. Teresa Alzamora

**Affiliations:** 1 Brainlab-Grup de Recerca en Neurociència Cognitiva, Departament de Psicologia Clínica i Psicobiologia, Institut de Neurociències, Universitat de Barcelona, 08035 Barcelona, Spain; iclemente@ub.edu; 2 Institut de Recerca Sant Joan de Déu, 08950 Esplugues de Llobregat, Spain; mmataro@ub.edu; 3 Unitat de Suport a la Recerca Metropolitana Nord, Fundació Institut Universitari per a la recerca a l’Atenció Primària de Salut Jordi Gol i Gurina (IDIAPJGol), 08303 Mataró, Spain; gpera@idiap.cat (G.P.); rosafores2011@gmail.com (R.F.); acostaga.mn.ics@gencat.cat (A.C.-G.); aheras.bnm.ics@gencat.cat (A.H.); noemilamonjavicente@gmail.com (N.L.-V.); ptoran.bnm.ics@gencat.cat (P.T.-M.); maiteal2007@gmail.com (M.T.A.); 4 Centre d’Atenció Primària Riu Nord-Riu Sud Santa Coloma de Gramenet, Direcció d’Atenció Primària Barcelonés Nord i Maresme, Institut Català de la Salut, 08921 Santa Coloma de Gramenet, Spain; 5 Centre d’Atenció Primària la Marina, Direcció d’Atenció Primària Barcelona Ciutat, Institut Català de la Salut, 08038 Barcelona, Spain; josemibaena@gmail.com; 6 Institut Universitari d’Investigació en Atenció Primària Jordi Gol (IDIAP Jordi Gol), 08007 Barcelona, Spain; 7 IGTP-HUGTP Biobank, Germans Trias i Pujol Research Institute (IGTP), 08916 Badalona, Spain; epedrosab@igtp.cat; 8 Departament de Psicologia Clínica i Psicobiologia, Institut de Neurociències, Universitat de Barcelona, 08035 Barcelona, Spain

**Keywords:** ankle brachial index, non-compressible artery disease, peripheral artery disease, 9p21.3, subclinical atherosclerosis

## Abstract

Peripheral artery disease (PAD) and non-compressible artery disease (NCAD) constitute predictors of subclinical atherosclerosis easily assessed through the ankle brachial index (ABI). Although both diseases show substantial genetic influences, few genetic association studies have focused on the ABI and PAD, and none have focused on NCAD. To overcome these limitations, we assessed the role of several candidate genes on the ABI, both in its continuous distribution and in the clinical manifestations associated to its extreme values: PAD and NCAD. We examined 13 candidate genomic regions in 1606 participants from the ARTPER study, a prospective population-based cohort, with the ABI assessed through ultrasonography. Association analyses were conducted independently for individuals with PAD (ABI < 0.9) or with NCAD (ABI > 1.4) vs. healthy participants. After including potential covariates and correction for multiple testing, minor alleles in the genetic markers rs10757278 and rs1333049, both in the 9p21.3 region, were significantly associated with a decreased risk of NCAD. Associations with the ABI showed limited support to these results. No significant associations were detected for PAD. The locus 9p21.3 constitutes the first genetic locus associated with NCAD, an assessment of subclinical atherosclerosis feasible for implementation in primary healthcare settings that has been systematically neglected from genetic studies.

## 1. Introduction

Atherosclerotic cardiovascular diseases (CVDs) continue to be the main cause of death in developed countries, although mortality associated to them has decreased in most regions of the world in the last decades [1]. In the United States, they are estimated to represent an economic burden of USD 11.3 trillion until 2050 [2]. Among other CVDs, peripheral artery disease (PAD) is a common manifestation of atherosclerosis characterized by a narrowing of peripheral arteries that increases cardiovascular and cerebrovascular morbid-mortality incidence [3]. Around the world, 202 million people have PAD. The prevalence of PAD [assessed as an ankle brachial index (ABI) < 0.9] ranges from 3.7 to 7% in the general population of countries with a low cardiovascular risk [4,5] with an overall increase during the last decades. Worldwide, the prevalence of this disease has increased more than 17% between 2010 and 2015 [6]. Conversely, a high ABI (ABI ≥ 1.4) result indicates non-compressible artery disease (NCAD), suggesting the presence of arterial calcification in the lower limbs [7], and is also associated with an increased risk of morbid-mortality [8,9,10]. The prevalence of NCAD is 6.2% in the same population to which the present study refers [11] and is twice as frequent in males as in females. In this sense, the ABI constitutes a predictor of subclinical atherosclerosis feasible to be systematically assessed in primary healthcare settings.

The main factors associated to an increased risk of PAD are environmental factors also associated to atherosclerosis, especially smoking, age, sex, type 2 diabetes, dyslipidemia, hypertension, or obesity, among others. However, several studies indicate a remarkable genetic component in the etiology of PAD. A family history of PAD and its clinical symptoms have been identified as independent risk factors for PAD [12,13], indicating a role for genetic or shared environmental factors. Moreover, twin studies have estimated that 58% and 48% of the observed variability in PAD and in ABI values, respectively, could be attributed to additive genetic effects [14,15]. Estimates of genetic influences for arterial calcification have yielded similar results, with heritability values ranging between 42 and 51% depending on the study and the type of calcification analyzed, although only abdominal aortic and coronary atherosclerotic calcification (CAC) have been examined (reviewed in [16]).

Despite this important genetic component, the specific genetic variants involved in pathological ABI values remain elusive. In contrast to thousands of association studies for other atherosclerotic pathologies, such as coronary artery disease or stroke, few reports on the ABI and PAD, and none on NCAD, have been published. For years, studies on the genetic bases of PAD used a candidate gene approach and focused on pathways relevant in atherosclerosis, such as lipid metabolism, endothelial function, or inflammation, among others. However, a meta-analysis from the candidate gene association resource (CARe) consortium for PAD and continuous ABI did not identify any robust association after examining 11 different studies [17]. With the advent of genotyping array technologies, several genome-wide association studies (GWASs) were conducted without a clear consensus on their findings. A meta-analysis of 21 GWASs published in 2012 only identified one genome-wide significant locus (9p21.3) with the ABI and none with categorical PAD [18]. There has not been much advance in the genetics of PAD until two recent efforts, which have shed some light on the genetic determinants of PAD by combining data from hundreds of thousands of individuals. Klarin and colleagues identified 19 loci associated with PAD in the Million Veteran Program (MVP) and the UK Biobank datasets [19], and a genome-wide meta-analysis just confirmed five of these associations in an independent sample of 449,548 individuals (12,086 PAD cases) [20]. One of these studies also included ABI data in the analyses, and locus 9p21.3 was the only genome-wide significant region associated with continuous ABI [19].

No previous studies have examined genetic determinants of NCAD assessed through the ABI. Moreover, genetic studies on the ABI have actively excluded individuals with values higher than 1.4 from their analyses (e.g., [17,18,19]). As for arterial calcification, some studies have focused on coronary artery calcification (CAC), another marker of subclinical atherosclerosis. In this sense, the most comprehensive meta-analysis of genome-wide association studies for CAC identified 11 independent risk loci as influencing CAC [21]. In addition, different association studies have screened the implication of specific candidate genes on arterial calcification revealing promising markers in specific pathways such as endothelial function, cell adhesion, and extracellular matrix remodeling (as a review, see [16]).

In the present study, we aimed at clarifying the relationship between variants in several candidate genes and differences in the ABI, both in its continuous distribution and in the clinical manifestations associated to its extreme values: PAD and NCAD. To do so, we explored fifteen genetic markers in candidate regions in the well-characterized subjects of the population-based Peripheral Arterial Disease (PERART/ARTPER) study. These candidate regions include regions previously associated to the ABI and genes associated to other atherosclerotic pathologies but not specifically tested for PAD or NCAD.

## 2. Materials and Methods

### 2.1. Subjects

A detailed description of the ARTPER study has been published previously [4,22]. Briefly, the ARTPER study is an ongoing prospective, multicenter, population-based cohort of 3786 subjects older than 49 years with the aim of determining the prevalence of peripheral artery disease and related cardiovascular risk factors in the general population. Individuals were recruited between 2006 and 2008, with periodic phone and in situ follow-up interviews. Later, between 2011–2012, 1810 participants provided a blood sample to be used in genetic studies. 

The study was approved by the local ethical review board (IDIAP Jordi Gol Foundation of Investigation in Primary Care (P11/61)), and all participants provided written informed consent. Moreover, the recommendations of the World Medical Association Declaration of Helsinki were followed.

### 2.2. Data Collection

The determination of the ankle brachial index (ABI) of the participants was carried under standardized conditions by two trained healthcare professionals using a standardized Doppler Ultrasonic device (Mini-Dopplex D 900-P, Huntleigh Healthcare, 8 MHz). A detailed description of the procedure has been previously published [4]. A threshold of an ABI < 0.9 was used to define PAD since it presents a high sensitivity and specificity for the detection of stenosis ≥50% in peripheral arteries [23,24]. Conversely, an ABI ≥ 1.4 was used to define the presence of calcification (NCAD) in the peripheral arteries [9].

A comprehensive questionnaire collected demographic, clinical, and lifestyle variables, including smoking status, physical activity, previous cardiovascular pathology (self-reported and in the clinical history), anthropometric measurements (height, weight, and waist circumference), hypertension, diabetes mellitus, and blood test results (total cholesterol, HDL-cholesterol, triglycerides, and glycemia), among others. The demographic and clinical characteristics of the final sample are described in Table 1.

### 2.3. Genetic Analyses

Genomic DNA was isolated from the whole peripheral blood by automated DNA purification based into M-PVA Magnetic Bead Technology using the Chemagic Magnetic Separation Module I instrument (Revvity, Inc., Waltham, MA, USA) at the IGTP-HUGTP Biobank (Spanish National Registry of Biobanks B.0000643) integrated in the Platform ISCIII Biobanks and Biomodels (PT20/00050). We determined DNA quality by the absorbance method. The 260/230 nm ratio was 1.7–2.1, and the 260/280 nm ratio was higher, 1.8, demonstrating a good DNA accuracy. Samples were processed following standard operating procedures with the appropriate approval of the Ethical and Scientific Committees.

Fifteen single-nucleotide polymorphisms (SNPs) in 13 different chromosomal regions were genotyped using KASPar assays, an allele-specific PCR technology, by an external genotyping core facility (Progenika Biopharma S.A., Derio, Spain). SNPs were selected based on two criteria: location in a gene or chromosomal region previously associated with PAD or with other related cardiovascular phenotypes and a minor allele frequency (MAF) higher than 10% in the Iberian population from the 1000 Genomes Project (except for the polymorphisms in the *APOE* gene) to maximize statistical power to detect genetic effects [25]. These included markers in gene regions involved in endothelial function, inflammation and immunity, or lipid metabolism, among other pathways. Details on the selected SNPs (identifier, chromosomal location, MAF, etc.) are described in Table 2.

### 2.4. Statistical Analyses

Quality control (QC) analyses were performed on the dataset. Forty-one negative controls and nine positive controls (i.e., sample duplicates) were included. Missingness by individual and by marker and deviations from Hardy–Weinberg equilibrium expectations were assessed to check data quality. After QC, the potential presence of population stratification effects between cases and controls was assessed through the calculation of the inflation factor (λ).

Association analyses were conducted by means of logistic regression models for binary traits (i.e., PAD and NCAD) and by linear regression models for continuous variables (i.e., ABI). All analyses compared, in different models, individuals with PAD or with NCAD vs. healthy participants. The analyses estimated effect sizes (odds ratio (OR) or β (β) for logistic and linear regressions, respectively) with their 95% confidence intervals under an additive genetic model. The analyses were conducted without covariates (unadjusted) and including all potential covariates identified in previous studies as influencing the ABI and PAD: sex, age, cardiovascular pathology, hypertension, diabetes, dyslipidemia, smoking, physical activity, central obesity, and abdominal obesity. Significant results were further evaluated under different models of genetic effect (i.e., dominant and recessive) in an attempt to clarify biological effects. To correct the rate of type I error, a false discovery rate (FDR) controlling procedure and a permutation test (1000 permutations) were implemented on significant results. The linkage disequilibrium was quantified between the markers in the 9p21.3 chromosomal region. All QC and association analyses were conducted using PLINK v.1.9 (https://www.cog-genomics.org/plink/1.9/ (accessed on 7 October 2019) [26]), specific for genetic association analyses.

Finally, we used the software QUANTO v.1.2.4 to estimate the minimum size of genetic effect that our cohort has statistical power to detect [27] (https://preventivemedicine.usc.edu/download-quanto/ (accessed on 15 September 2019)). We run post hoc power calculations based on our sample size and the minor allele frequency of the included SNPs and assuming additive effects, a statistical power of 80%, and a two-sided significance threshold of 0.05. Power calculations for the ABI as a quantitative outcome used a mean value of 1.039 and an SD of 0.130.

## 3. Results

### 3.1. Sample

From the original 1810 subject dataset, 58 samples were removed for low quality of genetic results, and 4 samples presented low call rate and were also removed. All genetic markers fit Hardy–Weinberg equilibrium expectations (*p* > 0.1), and all 50 controls (both positive and negative) were successfully identified. After quality control filters, genetic data for 1748 individuals were included, and the genotyping call rate was >99%. 

Subsequently, 119 subjects were removed because of discrepancies in data. Twenty-three subjects declared non-European ancestry and were not included to avoid potential population stratification effects in the study. Thus, the final sample included 1606 participants: 112 subjects with PAD (ABI < 0.9), 127 with arterial calcification (ABI > 1.4), and 1367 healthy controls (0.9 < ABI < 1.4) (Table 1). 

We did not detect signs of population stratification in our analyses, as estimated by inflation factors (λ) lower than 1.1 (λ of 1.00 and 1.08 for the adjusted and unadjusted analyses, respectively). Power calculations estimated that our cohort was powered to identify alleles than conferred increases in the risk of developing PAD or NCAD (GRR) higher than 1.40–1.43 and increases in ABI higher than 0.010–0.014 units, depending on the minor allele frequency of the analyzed SNPs.

### 3.2. Association Analyses

We did not find any significant association between the analyzed genetic variants and PAD (Table 2). Moreover, association analyses on the PAD severity, assessed as mild, moderate, or severe PAD (ABI values of 0.7–0.9, 0.4–0.7, and <0.4, respectively [13]), did not find significant associations. In contrast, polymorphisms in the 9p21.3 chromosomal region were significantly associated with the presence of calcified arteries in the lower limbs (Table 3). Minor alleles in SNPs rs10757278 and rs1333049 were significantly associated with a decreased risk of NCAD both in the unadjusted regression models and in the models adjusted for all covariates (*p* < 0.01 in all cases). Given the differences across groups for some environmental risk factors (Table 1), we further explored if the exclusion of some covariates, specifically cardiovascular pathology and hypertension, from the adjusted models modified the observations, but the association results remained very stable across adjusted, unadjusted, and different partially adjusted models.

In the post hoc analyses, we analyzed different genetic models to clarify the biological mechanism of the observed associations. Thus, we detected a higher degree of association between variants in 9p21.3 and NCAD under a recessive genetic model (results shown in Table 4), but there were not associations under a dominant model (*p* > 0.10). 

Individuals carrying two minor alleles in any of the SNPs explored in locus 9p21.3 had less than half the risk of presenting calcified arteries either under an unadjusted model (OR = 0.45; 95%CI = 0.26/0.77; *p* = 0.004) or after adjusting for all potential confounding variables (OR = 0.47; 95%CI = 0.27/0.81; *p* = 0.007). The unadjusted models resisted both a correction for multiple comparisons using an FDR method (*p* = 0.029) and a permutation test (*p* = 0.036–0.038), but the adjusted models only resisted the FDR control (*p* = 0.049) and not the permutation test (*p* = 0.085).

Association analyses of ABI as a continuous variable showed limited support to the results found in the analyses of binary traits. Under an additive model, no associations were identified between SNPs in the candidate genes and ABI neither when we analyzed individuals with PAD and controls nor among individuals with NCAD and controls (Supplementary Tables S1 and S2). We further conducted an association test between ABI and SNPs in the 9p21.3 region under a recessive genetic model since those were the analyses that showed the highest degree of association with arterial calcification (Table 4). Only SNP rs10757278 was associated with ABI at the standard nominal threshold (*p* = 0.040 in an unadjusted model and *p* = 0.053 in an adjusted model), with carriers of two copies of the minor allele (A) showing a lower ABI (β = −0.012; 95%CI: −0.024/−0.001). However, none of the comparisons held a correction for multiple comparisons or a permutation test (*p* > 0.3 in all cases).

## 4. Discussion

In this study, we analyzed fifteen polymorphic markers in 13 different genomic regions to identify genetic factors modulating the ABI, a well-known prognostic marker of CVD, and the risk to develop PAD or NCAD. We found that genetic variants at the 9p21.3 chromosomal region are significantly associated with an increased risk of NCAD in what constitutes, to our knowledge, the first genetic locus associated with arterial calcification assessed through ABI.

There is a broad consensus that ABI values higher than 1.4 are good predictors of subclinical atherosclerosis and cardiovascular risk assessment [7]. Other assessments of CV risk, such as CAC, may provide slightly better discrimination of risk, but its routine assessment is not feasible in primary healthcare settings since it is quantified through computed tomography [28,29]. Quick and non-invasive estimators of subclinical atherosclerosis, such as ABI, constitute practical alternatives to be implemented in primary care centers. However, clinical investigations on this complex phenotype have been limited compared with other CVD markers. Moreover, research on its genetic determinants has focused only on the lower tail of the distribution of ABI values, clinically associated to PAD, while systematically ignoring high values, associated to NCAD. 

In our study, we conducted separate analyses for both tails of the distribution of ABI values. Thus, we found a remarkable protective effect of the minor allele at both SNPs in the 9p21.3 region (rs10757278-A and rs1333049-G), which are in tight linkage disequilibrium. Homozygotes for the minor allele at any of these two polymorphisms, which account for almost a quarter of our population, showed a risk of developing NCAD that was 40–110% lower than carriers of major alleles (OR = 0.69 and 0.46 for the minor allele under additive and recessive models, respectively). 

Chromosome 9p21.3 harbors no known protein-coding genes, but it expresses a long noncoding RNA called *ANRIL* (antisense noncoding RNA in the *INK4* locus) that regulates genes in the atherogenic network [30]. Among other processes, *ANRIL* participates in the process of phenotypic switching of vascular smooth muscle cells to an adverse proliferative phenotype via microRNAs and the RAS/RAF/ERK signaling pathway (for a recent review, check [31]). The involvement of genetic variants at the 9p21.3 region in the pathophysiology of CVD was first identified by three independent GWASs in 2007 and has since been implicated in other CVD complications such as stroke or aneurysms. In this sense, our results extend to NCAD previous evidence on the implication of this chromosomal region in other endophenotypes of subclinical atherosclerosis, such as the ABI [18,19] and CAC [21]. This novel association, however, cannot be discussed with other reports on the genetics of NCAD, since the present work constitutes the first study published on the topic. So far studies have mostly focused on PAD as a binary outcome, and the few reports that have analyzed genetic determinants of the ABI as a continuous trait have actively excluded individuals with ABI > 1.4 from the analyses to remove heterogeneity from the analyses (e.g., [17,18,19]).

In contrast to coronary heart disease (CHD), very few genetic variants that influence susceptibility to PAD or ABI had been discovered until two recent genome-wide meta-analyses slightly broadened the list of associated loci [19,20]. Summarizing those and other previous works, the only locus that has been consistently associated with both PAD and ABI (limited to values lower than 1.4) is 9p21.3. In our study, we did not find any association between the markers in 9p21.3 or in any of the other 12 genomic regions analyzed and the risk to develop PAD or variation in ABI values. At most, a marginally significant association was observed between an SNP in the 9p21.3 region and ABI values among individuals with NCAD and controls but only under a recessive genetic model, the one with the strongest association with NCAD. Some of the difficulties identifying genetic factors associated to PAD and ABI might be attributed to the lower number of studies invested in the peripheral system and limited sample sizes, until some recent efforts. However, some authors have postulated that it might be also partly due to a greater degree of clinical and genetic heterogeneity in PAD [32]. In this sense, subdivision of precise phenotypes has been proposed as an approach to reduce heterogeneity and identify genetic markers that confer susceptibility to subclinical atherosclerosis [16]. The ABI can be considered an especially sensitive assessment of subclinical atherosclerosis since values at both extremes of its distribution are associated to different clinical manifestations: PAD and NCAD. This characteristic also implies that it needs special heed from an analytical point of view compared with other clinical measurements. Thus, a practical (but limited) approach has led researchers to remove high values from the analyses and focus only on PAD. Here we showed that separate analyses at both extremes of ABI values provide additional information on two different clinical entities related to subclinical atherosclerosis.

Other limitations of our study include the lack of a replication cohort to confirm our findings. Moreover, our analyses focused only on a restricted number of candidate genes instead of a genome-wide design, and thus, our conclusions were limited to a hypothesis-driven approach. Finally, our sample size had limited statistical power to detect small size effects, which tend to be the most common in genetic association studies of complex phenotypes, such as the ABI. Research in the field is starting to overcome this limitation by analyzing data from big cohorts, such as the UK Biobank or the MVP, but has also to be extended into generating joint consortia efforts, especially if multi-omics approaches are going to be implemented. In this sense, promising approaches to understand the pathophysiology of arterial calcification should consider the inclusion of system biology approaches combining at least epigenetic and genetic analyses. In the case of 9p21.3, for example, this locus exerts its functions through the expression of a non-coding RNA, which in turn is regulated by DNA methylation at the promoter region and by existing alleles [31]. Moreover, increasing evidence offers support for a relevant role of specific microRNAs in the pathophysiology, but also as diagnostic tools, of arterial calcification, PAD, and other atherosclerotic complications [33,34].

## Figures and Tables

**Table 1:** Demographic and clinical characteristics of participants. Data are presented as mean (SD) for continuous variables and as absolute (%) frequencies for binary variables.

	Controls (0.9 < ABI < 1.4)	PAD (ABI < 0.9)	NCAD (ABI ≥ 1.4)
N	1367	112	127
Age	67.9 (7.6)	72.5 (8.3) ***	70.2 (8.0) **
Male sex	567 (41%)	66 (59%) ***	86 (68%) ***
Cardiovascular pathology ^1^	146 (11%)	40 (36%) ***	17 (13%)
Hypertension	756 (55%)	86 (77%) ***	72 (57%)
Diabetes	276 (20%)	42 (38%) ***	36 (28%) *
Dyslipidemia	878 (64%)	84 (75%) *	81 (64%)
BMI	29.3 (4.6)	29.8 (4.87)	30.38 (4.7) *
Abdominal obesity ^2^	850 (63%)	71 (64%)	81 (64%)
Ever smoker	591 (43%)	73 (65%) ***	66 (52%)
Physical activity ^3^	6982 (4828)	5402 (3869) ***	7278 (5605)

^1^ Cardiovascular pathology defined as AIM, angina, stroke, or TIA. ^2^ Abdominal obesity defined as waist circumference ≥102/88 cm (male/female). ^3^ Physical activity defined as METS expenditure in 14 days performing leisure activity (walking, sport, shopping, cleaning, or gardening). *: *p* < 0.05; **: *p* < 0.01; ***: *p* < 0.001, in the comparisons with controls using Student’s *t* and chi-square tests for continuous and binary variables, respectively.

**Table 2:** Logistic regression results on PAD. Adjusted models included as covariates: age, sex, cardiovascular pathology, hypertension, diabetes, dyslipidemia, smoking, physical activity, central obesity, and abdominal obesity. A1/A2: minor/major allele; MAF: minor allele frequency; L95/U95: lower/upper bounds of the 95% confidence interval. Analyses were conducted under an additive model.

							Unadjusted Model					Adjusted Model				
Gene/Locus	Chr	SNP	Position	A1	A2	MAF	n	OR	L95	U95	*p*	n	OR	L95	U95	*p*
*TNFRSF9*	1	rs2453021	7929506	A	G	0.36	1473	0.97	0.72	1.29	0.809	1388	1.11	0.81	1.51	0.507
*MTHFR*	1	rs1801133	11796321	T	C	0.41	1476	0.97	0.73	1.28	0.819	1390	0.81	0.60	1.11	0.190
*OSBPL10*	3	rs1902341	31754078	A	G	0.42	1474	0.97	0.73	1.28	0.808	1388	0.98	0.73	1.33	0.915
*VEGF-A*	6	rs699947	43768652	A	C	0.47	1477	0.81	0.62	1.07	0.134	1391	0.82	0.61	1.10	0.176
*IL6*	7	rs1800795	22727026	C	G	0.35	1472	1.18	0.88	1.56	0.266	1387	1.17	0.86	1.59	0.317
9p21.3	9	rs10757278	22124478	A	G	0.48	1451	0.85	0.64	1.11	0.228	1366	0.91	0.68	1.22	0.516
9p21.3	9	rs1333049	22125504	G	C	0.48	1473	0.82	0.63	1.08	0.155	1387	0.86	0.64	1.16	0.330
*BDNF*	11	rs6265	27658369	T	C	0.22	1478	1.01	0.73	1.41	0.951	1392	1.04	0.73	1.48	0.845
*MMP1*	11	rs1799750	102799765	G	T	0.50	1470	1.03	0.79	1.36	0.822	1384	1.07	0.80	1.45	0.640
*MMP3*	11	rs35068180	102845216	C	A	0.47	1467	0.81	0.62	1.07	0.144	1382	0.79	0.58	1.07	0.126
*CCL2*	17	rs1024611	34252769	G	A	0.23	1471	1.15	0.84	1.58	0.381	1386	1.10	0.78	1.55	0.573
*CCL5*	17	rs2107538	35880776	T	C	0.17	1476	0.94	0.65	1.37	0.759	1390	0.94	0.63	1.40	0.772
*ICAM-1*	19	rs5498	10285007	G	A	0.50	1474	0.79	0.60	1.04	0.093	1388	0.78	0.57	1.06	0.107
*APOE*	19	rs429358	44908684	C	T	0.10	1465	0.79	0.48	1.30	0.357	1380	0.73	0.43	1.25	0.252
*APOE*	19	rs7412	44908822	T	C	0.05	1473	0.77	0.39	1.51	0.442	1387	0.81	0.39	1.69	0.568

**Table 3:** Logistic regression results on NCAD. Adjusted models included as covariates: age, sex, cardiovascular pathology, hypertension, diabetes, dyslipidemia, smoking, physical activity, central obesity, and abdominal obesity. A1/A2: minor/major allele; L95/U95: lower/upper bounds of the 95% confidence interval. Analyses were conducted under an additive model.

				Unadjusted Model					Adjusted Model				
Gene/Locus	SNP	A1	A2	n	OR	L95	U95	*p*	n	OR	L95	U95	*p*
*TNFRSF9*	rs2453021	A	G	1486	1.02	0.77	1.33	0.912	1467	1.06	0.80	1.41	0.700
*MTHFR*	rs1801133	T	C	1490	0.93	0.71	1.22	0.602	1469	0.95	0.72	1.24	0.690
*OSBPL10*	rs1902341	A	G	1488	1.04	0.80	1.36	0.761	1467	0.99	0.75	1.30	0.934
*VEGF-A*	rs699947	A	C	1492	0.92	0.72	1.19	0.536	1471	0.95	0.73	1.24	0.725
*IL6*	rs1800795	C	G	1486	0.91	0.68	1.20	0.483	1465	0.92	0.69	1.23	0.560
9p21.3	rs10757278	A	G	1467	0.70	0.54	0.91	0.007	1446	0.69	0.53	0.91	0.008
9p21.3	rs1333049	G	C	1487	0.69	0.53	0.90	0.006	1466	0.69	0.53	0.91	0.009
*BDNF*	rs6265	T	C	1493	1.24	0.92	1.67	0.157	1472	1.20	0.88	1.63	0.261
*MMP1*	rs1799750	G	-	1485	1.27	0.98	1.64	0.074	1464	1.28	0.98	1.68	0.068
*MMP3*	rs35068180	-	A	1481	1.02	0.79	1.33	0.870	1461	0.98	0.74	1.28	0.861
*CCL2*	rs1024611	G	A	1485	0.81	0.59	1.12	0.208	1464	0.83	0.59	1.16	0.269
*CCL5*	rs2107538	T	C	1492	0.94	0.66	1.32	0.705	1471	0.94	0.65	1.34	0.713
*ICAM-1*	rs5498	G	A	1489	0.85	0.65	1.10	0.223	1468	0.84	0.64	1.10	0.201
*APOE*	rs429358	C	T	1481	0.65	0.39	1.07	0.091	1460	0.66	0.40	1.11	0.116
*APOE*	rs7412	T	C	1488	0.83	0.44	1.54	0.544	1467	0.84	0.44	1.62	0.598

**Table 4:** Post hoc analyses under a recessive genetic model of associations between 9p21.3 markers and NCAD and ABI between NCAD and controls. Adjusted models included as covariates: age, sex, cardiovascular pathology, hypertension, diabetes, dyslipidemia, smoking, physical activity, central obesity, and abdominal obesity. A1/A2: minor/major allele; MAF: minor allele frequency; L95/U95: lower/upper bounds of the 95% confidence interval.

**NCAD**					**Unadjusted Model**					**Adjusted Model**				
**Gene/Locus**	**SNP**	**A1**	**A2**	**MAF**	**n**	**OR**	**L95**	**U95**	***p***	**n**	**OR**	**L95**	**U95**	***p***
9p21.3	rs10757278	A	G	0.48	1467	0.45	0.26	0.77	**0.004**	1446	0.47	0.27	0.81	**0.007**
9p21.3	rs1333049	G	C	0.48	1487	0.45	0.26	0.78	**0.004**	1466	0.47	0.27	0.81	**0.007**
**ABI**					**Unadjusted Model**					**Adjusted Model**				
**Gene/Locus**	**SNP**	**A1**	**A2**	**MAF**	**n**	**β**	**L95**	**U95**	***p***	**n**	**β**	**L95**	**U95**	***p***
9p21.3	rs10757278	A	G	0.48	1467	−0.012	−0.024	−0.001	**0.040**	1446	−0.012	−0.023	0.000	0.053
9p21.3	rs1333049	G	C	0.48	1487	−0.009	−0.020	0.002	0.110	1466	−0.008	−0.020	0.003	0.149

## Data Availability

The data presented in this study are available on request from the corresponding author. The data are not publicly available due to consent limitations.

## Supplementary Materials

The following supporting information can be downloaded at https://www.mdpi.com/article/10.3390/genes15010002/s1: Table S1: Linear regression results on the ankle brachial index (ABI) in PAD and control individuals; Table S2: Linear regression results on the ankle brachial index (ABI) in NCAD and controls.
